# Systematic literature review: should a bedtime snack be used to treat hyperglycemia in type 2 diabetes?

**DOI:** 10.1093/ajcn/nqac245

**Published:** 2022-09-09

**Authors:** Lauren A Roach, William Woolfe, Beenu Bastian, Elizabeth P Neale, Monique E Francois

**Affiliations:** School of Medical, Indigenous and Health Sciences, Faculty of Science, Medicine and Health University of Wollongong, Wollongong, New South Wales, Australia; Illawarra Health and Medical Research Institute, Wollongong, New South Wales, Australia; School of Medical, Indigenous and Health Sciences, Faculty of Science, Medicine and Health University of Wollongong, Wollongong, New South Wales, Australia; School of Medical, Indigenous and Health Sciences, Faculty of Science, Medicine and Health University of Wollongong, Wollongong, New South Wales, Australia; Illawarra Shoalhaven Local Health District, Diabetes Service, Wollongong, New South Wales, Australia; School of Medical, Indigenous and Health Sciences, Faculty of Science, Medicine and Health University of Wollongong, Wollongong, New South Wales, Australia; Illawarra Health and Medical Research Institute, Wollongong, New South Wales, Australia; School of Medical, Indigenous and Health Sciences, Faculty of Science, Medicine and Health University of Wollongong, Wollongong, New South Wales, Australia; Illawarra Health and Medical Research Institute, Wollongong, New South Wales, Australia

**Keywords:** type 2 diabetes, bedtime snack, fasting hyperglycemia, systematic review, glycemic control

## Abstract

**Background:**

Consuming a bedtime snack is often recommended for people with type 2 diabetes.

**Objective:**

This systematic review aims to evaluate the evidence from intervention studies to determine whether consuming a bedtime snack improves fasting hyperglycemia and/or overall glycemic control in individuals with type 2 diabetes.

**Methods:**

PubMed, Medline (EBSCO), Cochrane Library, and CINAHL Plus (EBSCO) databases were searched until 20 July, 2022. We included prospective studies in people with type 2 diabetes or prediabetes that included the intervention of a bedtime snack, consumed >30 min after dinner and <2 h before bed and reported glycemic outcomes.

**Results:**

The systematic review included 16 studies. There was no consistent relationship between consumption of a bedtime snack and improved glycemic control, especially when a no-snack control was included. Of the 4 studies that included the use of corn starch, a low dose seemed to have benefits over high-dose corn starch in terms of improved nocturnal and fasting glucose concentrations.

**Conclusions:**

Current advice to consume a bedtime snack is based on a limited number of intervention studies that often do not include a no-snack control, nor have used a feasible bedtime snack option that could be translated into everyday clinical practice. Further research is needed in type 2 diabetes patients treated with or without insulin. This review was registered at the Prospective Register of Systematic Reviews (PROSPERO) as CRD42020182523.

## Introduction

Type 2 diabetes poses a significant and growing health risk. It is estimated that in 2019, ∼9.3% of the population or 463 million adults had diabetes worldwide with ∼90% of these cases being type 2 diabetes (1). Type 2 diabetes is a well-documented risk factor for cardiovascular morbidity and mortality, reducing life expectancy by 5–15 y (2). Without appropriate management, this can lead to uncontrolled blood glucose concentrations and an increased risk of complications such as blindness, amputation, and CVD. HbA1c is an important indicator of glycemic control over the preceding 2–3 mo, comprising contributions from both postprandial and fasting glucose concentrations. Findings from several cohort studies have demonstrated a 2-fold greater risk of CVD with high HbA1c or fasting glucose concentrations (3–5). Accordingly, targeting HbA1c <53 mmol/mol (7.0%) and fasting glucose <7.2 mmol/L remain the cornerstone of diabetes treatment in order to reduce the risk of the above-mentioned diabetes complications (6, 7).

Fasting hyperglycemia is the term that describes elevated glucose concentrations upon waking after a nocturnal fast. In the morning, a cascade of gluconeogenic hormones are released as the body wakes, often causing a hyperglycemic event which if not regulated is known as the dawn phenomenon (8). Alternatively, another concept known as the Somogyi effect can occur, which describes the onset of hyperglycemia first induced by a hypoglycemic event that triggers high levels of gluconeogenesis to compensate for the dip in blood glucose (9). A potential strategy to reduce morning hyperglycemia is to consume a bedtime snack. Theoretically, ingestion of a snack before bedtime could mitigate fasting hyperglycemia by decreasing the nocturnal fasting window, reducing the gluconeogenic demands on the liver. Additionally, reducing the fasting window may elicit the second meal effect, which refers to the lasting impact a prior meal has on blood glucose concentrations following a subsequent meal (10). Furthermore, if ingestion of a bedtime snack results in fasting normoglycemia, overall glucose control and hence HbA1c could be improved.

However, despite the common recommendation given to patients with type 2 diabetes to consume a snack before bed (11), findings from clinical studies are inconclusive, with no study determining what the optimal composition of a bedtime snack may be. No study has systematically compared and appraised the evidence from intervention studies investigating the impact of a bedtime snack on blood glucose regulation in individuals with type 2 diabetes. The aim of this systematic review was to determine whether a bedtime snack, defined as any meal after dinner and within 2 h of bedtime, would attenuate fasting hyperglycemia and improve overall glucose control in individuals with type 2 diabetes.

## Methods

### Data sources and searches

This systematic review was reported in accordance with the Preferred Reporting Items for Systematic Reviews and Meta-Analyses (12). A protocol for the review was submitted to The International Prospective Register of Systematic Reviews (PROSPERO) (ID number: CRD42020182523) on 8 May, 2020.

A literature search was conducted across 4 databases (all years to 20 September, 2021). The following databases were used: PubMed, Medline (EBSCO), Cochrane Library, and CINAHL Plus (EBSCO), see **Supplemental Methods File 1** for search terms. Alternative spelling and truncations were included in the search strings, with both controlled vocabulary and free-text search terms used. Search terms were piloted in PubMed and then translated to other databases using Polyglot (13).

### Study selection and data extraction

To be included for review, studies were required to have a prospective study design and be published in English. Studies were also required to meet the following inclusion criteria: *1*) Include males and/or females diagnosed with type 2 diabetes, or classified as having insulin resistance or prediabetes based on Royal Australian College of General Practitioners (RACGP) and American Diabetes Association (ADA) diagnosis guidelines (6, 14) and over the age of 18 y; *2*) include the intervention of an oral bedtime snack (any product that had an energy content >0 kcal), consumed >30 min after dinner and <2 h before bed; *3*) assess outcomes related to glycemic control, including the measurement of glucose and insulin, and/or HbA1c.

In addition, the following exclusion criteria was applied:

*1*) All retrospective studies; *2*) studies including participants with type 1 or gestational diabetes; *3*) studies testing the effects of meals/snacks consumed >2 h prior to bed.

The online Covidence systematic review software (Veritas Health Innovation; www.covidence.org) was used to remove duplicates and then screen abstracts compared with the inclusion criteria above. Only full-text articles were included; conference abstracts were excluded but used to search for related full-text articles. Relevant articles were then retrieved for full-text review. All screening was conducted independently in duplicate (by author LAR, and either WW or BB). Two authors (MEF or EPN) then crosschecked the articles screened that met the inclusion criteria. Articles that did not clearly meet the inclusion or exclusion criteria were then discussed with all authors until a consensus was met. In the case that an article's population could not be completely identified as relevant, it was assumed the eligibility criteria was met. For example, if a study group had a mean HbA1c that identified as indicative of prediabetes or type 2 diabetes, the group was included even if the SD of the mean did not fall within the diagnostic range. The following data was extracted from each article: Citation, study quality, subject characteristics, intervention duration, intervention type, comparator, and study results. Extracted data was then collated in **Table 1**.

**TABLE 1:** Characteristics and outcomes of trials included in this review.

Citation	Age (y), BMI (kg/m^2^), sex	Intervention duration	Bedtime snack (kcal/g/kg carbohydrate per body weight)	Comparison group	Glycemic measures	Glycemic outcomes
Abbie, 2020 (25)	64 ± 6, 32 ± 6, 10 (F) 5 (M)	Crossover, 3 × 3-d intervention	1. 2 eggs (150 kcal) 2. Fruit-flavored Greek yogurt (150 kcal)	3. No bedtime snack (isocaloric dinner, so included the 150 kcal)	Continuous glucose monitoring. Plasma glucose and insulin	Mean 24h: No difference between conditions (*P* = 0.48) Mean 3-h postbreakfast: No significant difference between conditions (*P* = 0.19) Fasting blood glucose: Highest after yogurt (*P* < 0.05) compared with eggs. No differences between no snack and either snack intervention (*P* > 0.05) Nocturnal glucose: Highest after yogurt (*P* < 0.05) compared with eggs. No differences between no snack and either snack intervention (*P* > 0.36) Fasting insulin: Highest after yogurt (*P* < 0.05) compared with eggs. No differences between no snack and either snack intervention (*P* > 0.25)
Axelsen, 1997 (26)	55.3 ± 9.3, 29.0 ± 3.6, 1 (F) 9 (M)	Crossover, 3 × 2-d intervention with 7-d washout	1. Raw corn starch in low-sugar fruit juice (406 kcal, 106 g)^1^ 2. 3 wholegrain sandwiches (isolcaloric control meal) (406 kcal, 58 g)^1^ 3. 1 wholegrain sandwich (normal meal) (113 kcal, 17 g)^1^		Plasma glucose and insulin	Mean glucose: Lowest in the normal meal between 22:00–07:30 compared with both interventions (corn starch or isocaloric control meal) (both *P* < 0.05). No difference between corn starch and isocaloric control (*P* > 0.05). Significant rise in blood glucose concentration following nadir between 02:00–06:00 to 07:30 in isocaloric control and normal meal only (both *P* < 0.01) Glucose iAUC after breakfast: Lower in corn starch group compared with isocaloric control and normal meal (25% and 32%, respectively) (*P* < 0.05) Fasting glucose at 07:30: No difference between groups (*P* > 0.05) Nocturnal insulin AUC: Lowest after normal meal compared with both intervention groups (corn starch or isocaloric control) (*P* < 0.05). No difference between isocaloric control and normal meal between 02:00 and 07:30 (*P* > 0.05), 26% higher after corn starch at 02:00–07:30 compared with isocaloric control but NS (*P* > 0.05) Fasting insulin at 07:30: No significant difference between groups (*P* > 0.05) Insulin iAUC after breakfast: Similar between all groups (*P* > 0.05)
Axelsen, 1999 (27)	61 ± 9, 29.2 ± 5.0, 2 (F) 14 (M)	9 d (3 × 3-d, 5–10-day washout)	1. Corn starch (0.46 g/kg) 2. White bread (0.46 g/kg)	3. Pectin (0 kcal)	Plasma glucose and insulin	Fasting glucose: No difference (*P* > 0.05) Glucose iAUC after breakfast: 21% lower with corn starch compared with pectin (*P* < 0.05). No difference with white bread compared with pectin Blood glucose peak after breakfast: Lower with corn starch compared with pectin (*P* < 0.05). No difference with white bread compared with pectin Fasting insulin: No difference (*P* > 0.05) iAUC and peak insulin after breakfast: No difference (*P* > 0.05)
Axelsen, 2000 (28)	1. 56 ± 7, 29.2 ± 2.8, 3 (F) 11 (M) 2a. 61 ± 9, 28.8 ± 2.7, 5 (F) 7 (M) 2b. 60 ± 13, 28.8 ± 5.3, 5 (F) 7 (M)	1. Crossover, 2 × 7-wk intervention with 11-wk washout 2. Parallel 7-wk intervention	1. High-dose corn starch (0.59 g/kg) 2a. Low-dose corn starch (0.33 g/kg)	1a. Pectin placebo (0.10 g/kg) 2b. Pectin placebo (0.06 g/kg)	1. Plasma glucose and insulin, euglycemic hyperinsulinemic clamp technique, HbA1c 2. Plasma glucose and insulin, HbA1c	1. Nocturnal glucose: Increased glucose from 02:00 to 07:00 with corn starch (*P* < 0.01). Increase in glucose after nocturnal nadir in placebo and at baseline, but not in the corn starch group (*P* < 0.05) Fasting glucose: No change (*P* > 0.05) HbA1c: No change (*P* >0.05) Nocturnal insulin: Increase between 02:00 and 07:00 after corn starch (*P* < 0.01) Insulin sensitivity: No change (*P* > 0.05) Fasting insulin: No change (*P* > 0.05) 2. Fasting glucose: Lowest in corn starch group compared with placebo at 4 wk and 7 wk (both *P* < 0.05) HbA1c: No change (*P* > 0.05) Fasting insulin: No change (*P* > 0.05)
Beebe, 1990 (20)	50 ± 4, 35 ± 5, 2 (F) 4 (M)	Crossover, 3 × 26-h intervention with 2-wk washout	1. Snack 2.5 h after dinner (10% EI)^3^	2. No snack and 30:40:30 EI distribution 3. No snack and 10:20:70 EI distribution	Plasma glucose and insulin, insulin secretion rates determined by C-peptide distribution/clearance	Absolute glucose concentrations: Highest in snack group compared with comparators (*P* < 0.05) Late-night and early morning glucose (03:00–09:00): Raised in group 2 (*P* < 0.02), no difference in other groups Fasting glucose: No difference (*P* > 0.05) Total insulin secretion: Lower in snack group 1 compared with group 2 (*P* < 0.02) Fasting insulin: No difference (*P* > 0.05) Fasting insulin secretion rates: No difference (*P* > 0.05) Next morning insulin secretion rates (06:00–09:00): Highest in group 2 (*P* < 0.05)
Dyer-Parziale, 2001 (21)	61.9 ± 14.5, NR, 17 (F) 11 (M)	Crossover, 2 × 3-d intervention	1. Corn starch bar (160 cal, 31 g, 5 g corn starch) 2. Placebo bar (160 cal, 31 g)		Capillary glucose	Point glucose measures: No difference presnack. Lowest in corn starch group at midnight and before breakfast compared with placebo (both *P* < 0.0001)
Ilany, 2018 (22)	64 (54–73),NR, 7 (F) 4 (M)	Crossover, randomized to 2-d × no snack and remaining days snacks	1. 200 g cheese 2. 30 g nuts 3. 40 g extend bar	4. No bedtime snack	Continuous glucose monitoring	Fasting glucose: No difference between snack (combined) or no snack Overnight glucose: No difference between snack (combined) or no snack
Imai, 2017 (19)	70.3 ± 5.6, 22.8 ± 2.7, 8 (F) 8 (M)	Crossover, 5-d monitoring period, each intervention consumed once	1. Split dinner 18:00 (271 kcal 60.4 g) and 21:00 (353 kcal, 26.2 g)^1^	2. Dinner 18:00 (624 kcal, 86.6 g) ^1^ 3. Dinner 21:00 (624 kcal, 86.6 g)^1^	Continuous glucose monitoring, plasma glucose and insulin	Mean plasma glucose: No difference between split dinner and comparators iAUC glucose 23:00–08:00: Highest in late dinner compared with early and split dinner (both *P* < 0.01) Incremental glucose peak after dinner: Highest in late dinner compared with early (*P* < 0.001) and split dinner (*P* < 0.01) SD of plasma glucose: No difference (*P* > 0.05)
Imai, 2020 (18)	70.8 ± 1.9, 23.3 + 3.2, 3 (F) 5 (M)	Crossover, 3-d intervention, randomized to divided dinner or dinner at 21:00 on day 2 and 3	1. Split dinner 18:00 (355 kcal 78.9 g) and 21:00 (354 kcal, 25.3 g)^1^	2. Dinner 18:00 (709 kcal, 104.2 g)^1^ 3. Dinner 21:00 (709 kcal, 104.2 g)^1^	Plasma glucose and insulin	Incremental glucose: Highest in later dinner compared with 1st meal of split dinner at 30, 60, and 120 min (*P* < 0.01, *P* < 0.05, *P* < 0.01). Lowest 120 min after 2nd meal of split dinner compared with both comparison groups (both *P* < 0.01) iAUC 4-h glucose: Lower in split dinner compared with late dinner (*P* = 0.070). No difference between split and early dinner Incremental insulin: Highest in late dinner compared with 1st meal of split dinner at 60 and 120 min (both *P* < 0.05). Lowest 120 min after 2nd meal of split dinner compared with late dinner (*P* < 0.05) AUC 4-h insulin: Lower in split dinner compared with late dinner (*P* = 0.070). No difference between split and early dinner
Kahleova, 2014 (32)	59.4 ± 7.032.6 ± 4.925 (F) 29 (M)	Crossover trial, 12-wk intervention	1. 6 meals including a bedtime snack	2. 2 meals (breakfast and lunch) isocaloric to intervention 1 with the same calorie restriction as intervention 1 (500 kcal/d)	Plasma glucose and insulin. Insulin sensitivity. HbA1c	Fasting plasma glucose: decreased under both regimens (*P* < 0.001), more with intervention 2 (*P* = 0.004) Fasting immunoreactive insulin: decreased (*P* < 0.04) comparably with both regimens HbA1c: decreased (*P* < 0.001) comparably with both regimens Insulin sensitivity: Increased with both regimes (*P* < 0.001)
Kim, 2016 (29)^2^	55.2 ± 8.0, 23.7 ± 2.9,9 (F) 4 (M)	Crossover with 2 × 2-d intervention, 7-d washout	1. Fiber-enriched flakes (279 kcal, 43 g) 2. Cereal flakes (279 kcal, 44 g)		Plasma glucose and insulin	Fasting glucose: No difference (*P* > 0.05) Incremental glucose: No difference at 08:00. Lower in fiber group at 2-h postbreakfast (*P* < 0.05). Peak increment of glucose: Lowest in fiber group (*P* < 0.001) iAUC of glucose: Lowest in fiber group 08:00–12:00 (*P* < 0.001). No difference 12:00–16:00 (*P* > 0.05) Incremental and iAUC insulin: No difference (both *P* > 0.05)
Nakanishi, 2019 (23)	61.5 ± 11.5, 30.1 ± 3.8,2 (M)^4^	Crossover, 2 × 7-d intervention	1. nutrient-enriched BCAA (210 kcal)	2. No snack	Flash glucose monitoring	7-d mean glucose: Lowest in no snack group (*P* < 0.01)
Papakonstantinou, 2018 (24)	i. IGT-A: 43.8 (3.1), 32.6 ± 1.4, 10 (F) 5 (M) ii. IGT-B: 52.1 (2.7), 32.5 ± 1.2, 10 (F) 10 (M) iii. T2D: 51.7 (3.5), 32.2 ± 1.5, 5 (F) 7 (M)	Crossover, 2 × 12-wk intervention	1. Bedtime snack (<150 kcal, 10% daily carbohydrates)^3^	2. No bedtime snack^3^	Plasma glucose and insulin	Post-OGTT glucose: i., ii. No difference (*P* > 0.05) iii. Lowest in bedtime snack group at 90 min and 120 min compared with baseline (both *P* < 0.05) and compared with no snack at 60 min, 90 min, and 120 min (all *P* < 0.05) Fasting glucose: i, ii., iii. No difference (*P* > 0.05) iAUC of glucose: i., ii., iii. No difference (*P* > 0.05) HbA1c: i., ii. No difference (*P* > 0.05)iii. Lowest in bedtime snack group (*P* < 0.001) Post OGTT insulin: i. Lowest with bedtime snack group at 30 min compared with no snack and with baseline at 30 min and 60 min (all *P* < 0.05)ii., iii. No difference (*P* > 0.05) Fasting insulin: i., ii., iii. No difference (*P* > 0.05) iAUC of insulin: i., ii., iii. No difference (*P* > 0.05)
Sapp, 2021 (17)	42 ± 15, 28.3 ± 5.6, 25 (F) 25 (M)	Crossover, 6-wk intervention with 4-wk washout	28 g/d dry-roasted unsalted peanuts (164 kcal)	6 low-sodium wholegrain crackers and 1 slice low-fat cheese (165 kcal)	Plasma glucose and insulin	Fasting glucose: No difference within or between conditions (*P* > 0.05) Fasting insulin: No difference within or between conditions (*P* > 0.05)
Suzuki, 2010 (30)	i. Chronic hepatitis: 56 ± 14, 23.5 ± 3.7, 3 (F) 8 (M) ii. Liver cirrhosis: 66 ± 8, 22.9 ± 3.7, 15 (F) 21 (M)	14 d (2 × 7 d)	1. Bedtime snack (210 kcal, NR)	2. No bedtime snack	Plasma glucose and insulin	Incremental glucose: i. Lowest in bedtime snack group before and 2 h after dinner (both *P* < 0.05) ii. Lowest in bedtime snack group 2 h after breakfast, before lunch, and before dinner (all *P* < 0.05) Average glucose concentration s: i. No difference (*P* > 0.05) ii. Lowest in bedtime snack group (*P* < 0.05) Peak glucose concentration s: i., ii. No difference (*P* > 0.05) Daily total insulin secretion: i., ii. No change (*P* > 0.05)
White and Johnston, 2007 (31)	40–72, 29.1 ± 1.2, 7 (F) 4 (M)	Crossover, 2 × 2-d intervention with 3–5-d washout	1. 2 tbsp apple cider vinegar and 28 g cheese (50 kcal, 1 g) 2. 2 tbsp water and 28 g cheese (50 kcal, 1 g)		Capillary glucose	Fasting glucose: 4% decrease in vinegar group from baseline (*P* = 0.046). No change after water from baseline (*P* = 0.928)

BCAA, branched-chain amino acid; EI, energy intake; iAUC, incremental AUC; IGT, impaired glucose tolerance; NR, not reported; NS, not significant; T2D, type 2 diabetes.

1 Was part of a separate intervention comparing a high-fiber cereal to a conventional cereal when eaten for dinner, bedtime snack, and breakfast.

2 Amounts based on the study mean and not an individual basis.

3 Was part of a separate intervention comparing the effects of 6 compared with 3 eucaloric meal patterns.

4 Study included another group which did not fit the inclusion criteria.

### Quality assessment

Study quality was independently assessed by authors (LAR, and either WW or BB) using the American Dietetic Association Appraisal tool and National Health and Medical Research Council (NHMRC) level of evidence matrix (15, 16). The checklist examines the quality of the study by considering the relevance and validity of the design. In particular, the tool explores generalizability, practicality, clarity, selection bias, appropriateness of comparator, reporting bias, blinding, validity of measurements, appropriateness of statistical methods, and potential of bias from funding source(s).

## Results

**FIGURE 1:**
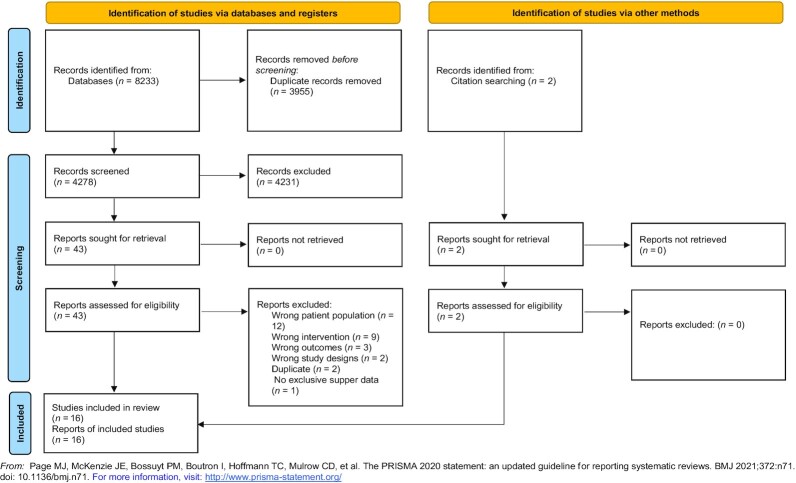
Flow diagram for the selection and identification of articles included in this systematic review on the inclusion of a bedtime snack for the treatment and management of hyperglycemia in people with type 2 diabetes and prediabetes.

A total of 8233 articles were identified, after removing duplicates 4278 titles and corresponding abstracts were screened. From these, 43 full-text articles were then assessed against the eligibility criteria for inclusion. A total of 16 studies were deemed eligible and included in this review (**Figure 1**).

Studies included in this review investigated the impact of a bedtime snack on glycemic control in people with type 2 diabetes or prediabetes (Table 1). The average age of the participants studied ranged from 42 (17) to 71 y (18). Participants had a BMI ranging from 22.8 (19) to 35 kg/m^2^ (20), with 2 studies not recording BMI (21, 22). A single study only included males (for the subset who had diabetes) (23), whereas all other studies included male and female participants (17–22, 24–31). A total of 13 studies included participants taking diabetes medications (18–23, 25–29, 31, 32), of these, only 1 study included participants taking insulin (21). Of the 13 studies, participants prescribed metformin only was included in 2 studies (22, 29), participants prescribed metformin or sulfonylureas were included in 3 studies (26–28), whereas a mix of diabetic medications [dipeptidyl peptidase-4 (DPP4) inhibitors, sodium-glucose co-transport 2 (SGLT2) inhibitors, α-glucosidase inhibitors, thiazolidinedione, glinides] were reported in 3 studies (18, 25, 32) and lastly, 3 studies did not report the class of diabetic medications the participants were prescribed (19, 23, 31). The remaining 3 studies, included participants who were not prescribed any diabetic medication (17, 24, 30). The length of intervention varied, ranging from 1 d (18, 19) to 12 wk (24, 32). Interventions included a range of different bedtime snacks: 5 studies used standard food items (e.g. eggs, bread, cheese, nuts, or yogurt) (17, 22, 25–27); 4 included a corn-starch-based product (21, 26–28); 2 adopted a split dinner approach in which dinner was split into 2 heterocaloric meals separated by a specified time (18, 19); 1 used fiber-enriched cereal (29); 1 used nutrient-enriched BCAAs (23); 1 used vinegar and cheese (31); lastly, 3 studies used a varied meal distribution approach with standard food items containing all macronutrients used as the bedtime snack (20, 24, 30). One study looked at hypocaloric meals given as 2 meals versus 6 meals on glycemic measures (32). The majority of articles used the absence of a meal/snack as a comparator (18–20, 22–25, 30). Other studies used: A wholegrain sandwich (26), a pectin solution (27, 28), non corn-starch bar (21), conventional cereal (29), cheese, nuts, and a snack bar (22), water and cheese (31), or cheese and wholegrain crackers (17). All studies measured ≥1 measurement of blood glucose concentration, including fasting glucose and incremental glucose AUC (iAUC) (17–32), 12 of the 16 studies examined plasma insulin concentration (17–20, 24–30, 32) and 3 of the 16 studies analyzed HbA1c (24, 28, 32). Results are described in greater detail below.

### Interventions that were compared with a no-snack or calorie-empty control

#### Standard food interventions

A total of 4 articles with 80 participants in total investigated a bedtime snack in comparison to no-snack or calorie-empty control, few benefits to glucose measures were reported. Abbie et al. found no improvements in fasting, mean, nocturnal, and 3-h postbreakfast plasma glucose after consuming an egg or yogurt snack 30–45 min prior to bed when compared with a no-snack control (25). Furthermore, no difference in fasting plasma insulin was observed after having a snack (yogurt or egg) compared with not having a bedtime snack (25). From the 11 participants with type 2 diabetes and impaired fasting glucose in the study by Ilany et al., overall, there was no difference in fasting blood glucose or overnight glucose when a bedtime snack was consumed compared with no snack. From this study, there were 2 participants who had consistently lower fasting glucose as well as lower AUC at night with all bedtime snacks (either fat, protein, or carbohydrate based), compared with no snack (22). These study results together demonstrate that having a bedtime snack compared with no bedtime snack did not improve glycemic outcomes in the study population.

Two studies by Axelsen et al. compared ingestion of corn starch to a calorie-empty control (27, 28). Ingestion of a high-dose corn starch product (0.55 g/kg) at 22:00 resulted in significantly higher nocturnal blood glucose (2.1 ± 0.4 mmol/L increase, mean ± SEM) between 02:00 and 07:00, when compared with a calorie-empty control (28) and no observed improvement in HbA1c after 7 wk of consumption (28). However, fasting glucose was significantly reduced from a low-dose corn starch treatment (0.30 g/kg) compared with control at both 4 and 7 wk by 0.8 ± 0.4 mmol/L and 0.9 ± 0.4 mmol/L, respectively (mean ± SEM) (28). Axelsen et al. reported a 21% reduction in postprandial blood glucose concentration following breakfast when a moderate dose of corn starch (0.46 g/kg) was consumed the night before compared with a calorie-empty control (27).

Axelsen et al. noted that neither a high nor low dose of corn starch improved fasting insulin compared with calorie-empty controls. Additionally, high-dose corn starch had no impact on insulin sensitivity determined by euglycemic hyperinsulinemic clamp, but did significantly increase nocturnal insulin between 02:00 and 07:00 by 5.1 ± 1.6 mU/L compared with the control (mean ± SEM) (28). Another study found that 4 h iAUC and peak insulin following a standard breakfast did not differ after moderate corn starch ingestion the previous night before bed compared with a pectin control (27).

#### Interventions of split meals and altered meal distribution

Six studies with a total of 167 participants investigated either split meals or altered meal distribution. Dividing the main nightly meal into 2 smaller meals (1 consumed before bedtime) has been proposed to reduce postprandial hyperglycemia in people with type 2 diabetes. Both studies that implemented a split dinner (18:00 and 21:00) found that the intervention was superior to an isocaloric late dinner (21:00); with significantly reduced nocturnal plasma glucose iAUC between 23:00 and 08:00 (142 ± 60 compared with 644 ± 156 mmol/L × min, mean ± SEM), reduced postprandial incremental plasma glucose peak following dinner (3.75 ± 0.58 compared with 6.78 ± 0.79 mmol/L, mean ± SEM) (19), and lower iAUC plasma glucose at 30-, 60-, and 120-min postdinner (18). However, when compared with an early dinner (18:00) a split dinner only resulted in significant reduction in postprandial glucose concentration at 120 min (18). An early dinner at 18:00 was superior to a late dinner at 21:00 as the increase in plasma glucose was significantly different at 60-min postdinner (2.03 ± 0.51 compared with 4.45 ± 0.93 mmol/L, mean ± SEM), and 120-min postdinner (3.35 ± 0.84 compared with 6.75 ± 1.22 mmol/L, mean ± SEM) (18). In addition, the split dinner intervention resulted in a trend of reduced 2-h insulin iAUC (552 ± 114 compared with 772 ± 104 µU/mL, mean ± SEM) (*P* = 0.070) and significantly reduced postprandial incremental insulin at 60 and 120 min (60 min, 1.80 ± 1.50 compared with 7.58 ± 1.54 µU/mL, 120 min, 2.36 ± 2.24 compared with 11.3 ± 1.97 µU/mL, mean ± SEM) (both *P* <0.05) in comparison to a late dinner. There was no significant difference between a split dinner and an early dinner for all measured insulin outcomes (18).

In addition, altering the distribution of daily calories (and carbohydrate) to smaller more frequent meals is another strategy to attenuate hyperglycemia. Interventions that employed a meal distribution approach, found having 6 meals over the day (including a bedtime snack) compared with isocaloric 3 main meals with no bedtime snack had no effect on fasting plasma glucose, insulin, or serum insulin (20, 24). However, for people with impaired glucose tolerance, consuming 6 meals (including a bedtime snack) for 12 wk significantly lowered plasma insulin concentrations at 30-min post-OGTT compared with consuming 3 meals per day without a bedtime snack (24) and for those with type 2 diabetes, improvements in HbA1c, and plasma glucose concentrations at 60-, 90-, and 120-min post-OGTT were observed after consuming 6 meals compared with 3 (24). In contrast, average daily glucose was 10–15% higher after consuming 3 meals and 3 snacks (including a bedtime snack) when compared with either 3 standard isocaloric meals or a large dinner in a small crossover study (20); furthermore, the large dinner intervention resulted in a lower mean daily plasma glucose and insulin secretory rate than the 6 meals with a bedtime snack (20). However, there was no difference in plasma glucose the following morning after a bedtime snack, large dinner, or standard dinner (20). Similarly, Kahleova et al. found that fasting insulin improved when patients with type 2 diabetes had 2 meals of breakfast and lunch rather than an isocaloric 6 meals including dinner and a bedtime snack with the same restriction of 500 kcal/d, however, both groups had similar improvements in HbA1c (32).

A late evening snack at 22:00, of 200 kcal, was tested in a population of liver disease patients following a period of 3 meals per day with no bedtime snack (30). After consuming the late evening snack for 7 d (overall daily energy intake isocaloric), patients with hepatitis and elevated fasting glucose had significantly improved plasma glucose concentrations before dinner and 2-h postdinner, and patients with liver cirrhosis had improved plasma glucose concentrations 2-h postbreakfast, before lunch, and before dinner when compared with the period of 3 meals with no bedtime snack (30). The late evening snack had no impact on insulin resistance (determined by HOMA-IR) for either of the groups (30).

#### Nutritional supplementation

A single study with 13 participants investigated the effect of a nutrient-enriched BCAA supplement administered at bedtime in a drink, for 7 d in people with cirrhosis. In a subset of the group (*n* = 2) who met the criteria for diabetes (for nocturnal glucose) at baseline, this bedtime snack had a detrimental effect on 7-d average glucose concentrations measured using a flash glucose monitoring system, when compared with a no snack period (200.9 ± 59.7 compared with 153.6 ± 43.3 mg/dL, mean ± SEM). For those who did not have elevated nocturnal blood glucose at baseline, there was no significant difference between the snack and no snack (23).

### Interventions compared with bedtime snack comparators

A total of 6 studies with 127 participants compared different bedtime snacks. Comparing snacks of differing nutrient composition and volume is important to determine the optimal type of bedtime snack for managing hyperglycemia. For example, Dyer-Parziale et al., found that a corn starch bar resulted in significantly lower capillary glucose concentrations when measured at midnight and before breakfast compared with an isocaloric, carbohydrate-matched (30 g each) standard cereal bar (21). Alternatively, Axelsen et al., concluded that following ingestion of a large dose of corn starch in low-sugar fruit juice (100 g corn starch, providing 106 g carbohydrate), mean nocturnal plasma glucose was significantly higher compared with a lower energy wholegrain sandwich (providing 17 g carbohydrate) but not different from 3 wholegrain sandwiches (providing 58 g carbohydrate, chosen as an isocaloric control to the large dose of corn starch). Consuming 3 open-faced wholegrain sandwiches (58 g carbohydrate) resulted in significantly higher nocturnal glucose (22:00 to 07:30) compared with those who consumed 1 open-faced wholegrain sandwich (17 g carbohydrate) before bed (9.0 ± 0.5 compared with 7.4 ± 0.3 mmol/L, mean ± SEM). Both sandwich interventions experienced a dip in nocturnal glucose and a subsequent rise in fasting concentrations, whereas the corn starch intervention did not dip overnight, the 1 sandwich group rose on average by 1.2 ± 0.2 mmol/L whereas the 3 sandwich group rose by 0.6 ± 0.2 mmol/L (26). In addition, following breakfast the next day, postprandial glucose was lowest in the corn starch group by 25% and 32% compared with the 3 sandwich and 1 sandwich groups, respectively (26). Lastly, insulin AUC for the whole night was significantly lower for 1 sandwich compared with 3 sandwiches or the corn starch treatment (26). There was no difference in fasting insulin at 07:30 or iAUC after breakfast between groups (26).

One article demonstrated that consuming fiber-enriched cereal for dinner, a bedtime snack, and breakfast was superior to a conventional isocaloric cereal (consumed at the same timepoints) in blunting 2-h postprandial breakfast plasma glucose (198.5 ± 12.8 compared with 245.9 ± 15.2 mg/dL, mean ± SEM), peak increment of plasma glucose (101.8 ± 9.1 compared with 140.3 ± 14.3 mg/dL, mean ± SEM), and iAUC of 0–4 h postbreakfast (202.0 ± 23.9 compared with 301.1 ± 37.7 mg hr/mL, mean ± SEM). There was no difference in fasting plasma glucose after consuming the 2 different cereals, and no additional effects of the fiber-enriched cereal were observed within the 4 h after lunch; lastly, no differences in insulin measures were observed between the 2 cereals (29).

White and Johnston noted that a bedtime snack of cheese and vinegar decreased fasting capillary glucose by 4% (0.26 mmol/L) compared with baseline. Whereas cheese and water had no significant effect when also measured against baseline. Overall, there was a significant time-by-treatment effect (*P* = 0.03). However, a stronger effect was seen in individuals with higher glucose concentrations at baseline (>7.2 mmol/L), in these individuals (*n* = 5) fasting capillary glucose was reduced by 6% after the vinegar and cheese treatment (31).

Abbie et al. compared 2 eggs to a calorie- and protein-matched flavored Greek yogurt consumed before bedtime in an acute crossover study. The egg snack resulted in significantly lower fasting glucose (7.2 ± 0.2 compared with 7.6 ± 0.2 mmol/L, mean ± SD) and insulin (111 ± 52 compared with 128 ± 56 pmol/L, mean ± SD) the following morning and lower nocturnal glucose (7.6 ± 0.2 compared with 8.2 ± 0.3 mmol/L, mean ± SD) compared with the yogurt snack (25). Lastly, Sapp et al. found no significant effect on fasting glucose or insulin in those with elevated fasting glucose who consumed either peanuts or isocaloric wholegrain crackers and cheese as a bedtime snack for 6 wk in a crossover trial (17). In addition, neither the peanuts nor the cheese and crackers showed any improvement in fasting glucose or insulin when compared with baseline measures (17).

### Study quality

Study quality is reported in **Table 2**. A total of 9 studies were assigned a neutral rating, of these, 2 studies lacked relevance and validity (29, 31) whereas the remaining 7 lacked validity only (20, 21, 23, 26–28, 30). Reasons for lacking relevance included inadequate measures of outcomes and poor feasibility of the intervention. Validity criteria that commonly resulted in studies being categorized as neutral included: insufficient eligibility criteria, insufficient information about the randomization process, lack of blinding where appropriate, inability to identify limitations and inadequate measurement of outcomes. Six studies were assigned a positive rating (17–19, 24, 25, 32). Last, 1 study received a negative rating due to lacking validity (22).

**TABLE 2:** Level of evidence and study quality of trials included in this review.

Citation	NHMRC level of evidence	American Dietetic Association study quality
Abbie, 2020 (25)	II	+
Axelsen, 1997 (26)	II	∅
Axelsen, 1999 (27)	II	∅
Axelsen, 2000 (28)^1^	1. II	∅
	2. II	∅
Beebe 1990, (20)	III-1^2^	∅
Dyer-Parziale, 2001 (21)	II	∅
Ilany, 2018 (22)	III-3	–
Imai, 2017 (19)	II	+
Imai, 2020 (18)	II	+
Kahleova, 2014 (32)	II	+
Kim, 2016 (29)	II	∅
Nakanishi, 2019 (23)	III-1^2^	∅
Papakonstantinou, 2018 (24)	II	+
Sapp, 2021 (17)	II	+
Suzuki, 2010 (30)	III-1^2^	∅
White and Johnston, 2007 (31)	II	∅

NHMRC, National Health and Medical Research Council.

+ Positive quality.

– Negative quality.

∅ Neutral quality.

1 Axelsen 2000 contains 2 separate studies and so these were graded separately.

2 Articles that did not specifically state the protocol for randomization were allocated a rating of III-1.

## Discussion

This is the first study to systematically review evidence regarding the impact of consuming a bedtime snack on glycemic control in people with prediabetes and type 2 diabetes. Overall, findings indicate no benefit of consuming a bedtime snack over a no-snack control for improving measures of glycemic control, including fasting and nocturnal glucose concentrations (20, 21, 24–28). Studies that compared different bedtime snack compositions found that a resistant starch snack or a low-carbohydrate bedtime snack resulted in better glycemic profiles than higher carbohydrate snack comparators (21, 25). Of note, carbohydrate-containing bedtime snacks (even corn starch), in general, tended to cause a rise in postprandial glucose concentrations before bed and nocturnal glucose concentrations. Only 3 studies utilized standard food interventions. Of the interventions, resistant starch appears to be the most promising, however, investigations of other resistant starches and of whole standard food items that are low in carbohydrate are limited.

Carbohydrate-rich foods are often chosen for bedtime snacks as traditionally it is recommended to distribute carbohydrates over the day to control blood glucose concentrations (33). A bedtime snack of corn starch was the most common snack investigated due to corn starch being a complex carbohydrate with a lengthy digestibility of ≤6–9 h, slowly releasing glucose into the bloodstream and modestly increasing insulin to reduce hepatic glucose production (34, 35). However, our findings have shown that the higher doses of corn starch often resulted in increased nocturnal glucose compared with a sandwich or a placebo (26, 28), and resulted in no improvement in fasting glucose concentrations compared with sandwiches or placebo (26, 27). Long-term benefits of corn starch at bedtime were observed by Axelsen et al., with reduced fasting plasma glucose concentrations at 4 and 7 wk, compared with placebo, it is important to note this was from a lower dose of corn starch (28). There was no observed improvement in HbA1c at 7 wk, however, HbA1c likely requires a longer period than 7 wk to reflect changes in glycemic control (36). Furthermore, a corn starch bar at bedtime resulted in reductions in capillary glucose measures at midnight and fasting concentrations compared with a carbohydrate-matched bar without corn starch (21). Corn starch in low doses may have some benefit to fasting and postprandial glucose concentrations, however, the nocturnal response to the lower doses of corn starch has not been investigated; furthermore, consuming corn starch prior to bed is not a palatable and therefore feasible option for many. Yogurt is another carbohydrate-based food which has been investigated as a snack prior to bed. A yogurt snack (providing 24.38 g of carbohydrate) resulted in no difference to fasting plasma glucose or glucose control (determined by continuous glucose monitoring) when compared with having no snack before bed in an acute study by Abbie et al. (25).

A protein-based snack may be recommended before bed as higher protein diets may exert some benefit to glycemic control in people with type 2 diabetes, such as reduced HbA1c (37). Abbie et al., found a snack of 2 cooked eggs consumed before bed significantly improved fasting plasma glucose and insulin and nocturnal glucose concentrations when compared with a carbohydrate-based yogurt snack (25). However, the egg snack was not superior to a no-snack condition, suggesting if a snack is preferred by the patient, a protein-based snack may be a better option but no better than not consuming a snack at all. Conversely, when Nakanishi et al., investigated the use of a BCAA supplement as a late evening snack in cirrhosis patients, there was no benefit to glucose metabolism compared with not consuming a bedtime snack (23). This is surprising as BCAAs have been shown to promote insulin stimulation and glucose utilization (38). In particular, individuals with a nocturnal blood glucose concentration ≥200 mg/dL (criteria for diabetes diagnosis) had worsening average blood glucose (determined every 15 min by continuous glucose monitoring) after the BCAA bedtime snack, however, this subgroup only had a sample of *n* = 2 and given their pathogenesis, the finding lacks generalizability to people with type 2 diabetes alone (23).

The consumption of fat has been shown to delay gastric emptying in healthy people (39, 40) and people with type 2 diabetes, which can improve the postprandial glycemic response (41). Therefore, a fat-based snack before bedtime may be preferable to a carbohydrate-based snack for nocturnal glycemic control. Indeed, Abbie et al., found improvements to nocturnal and fasting glucose following the consumption of 2 boiled eggs, compared with a carbohydrate-based low-fat yogurt snack prior to bed (25). Furthermore, White & Johnston reported that a bedtime snack of vinegar and cheese reduced fasting glucose by 4%, whereas cheese alone did not result in a significant change in fasting glucose. However, the authors speculate that a synergistic effect of the cheese and vinegar may have occurred, once again this study was small in size and used capillary glucose as the only outcome measure and so must be interpreted with caution (31).

The consumption of dietary fiber has reported long-term benefits to people with type 2 diabetes including reduced HbA1c and fasting plasma glucose (42). The consumption of fiber-rich foods as a bedtime snack may therefore reduce hyperglycemia through their ability to slow gastric emptying (42). In addition, dietary fiber produces SCFAs through fermentation in the colon, these SCFAs increase GLP-1 secretion, which has been shown to enhance glucose-stimulated insulin secretion (43). A dietary fiber-enriched cereal consumed at dinner, as a bedtime snack, and then for breakfast the next morning resulted in a better glycemic response to breakfast compared with a standard cereal (29). It is unclear if any of the improved glucose responses was due to cereal consumed at bedtime as the same cereal was consumed at breakfast and there was no difference in fasting glucose concentrations between cereal types. However, dietary fiber can remain in the digestive tract overnight and so may have exerted an effect the next morning (44).

Overall, the clinical evidence included in this systematic review is limited by several factors, mainly as we were unable to conduct a meta-analysis we are unable to comment on the average effect of bedtime snacks on glycemic control and whether a specific kind of snack is effective for improving glycemic control. Included in this review were studies with other comorbidities such as hepatitis and cirrhosis, with findings lacking generalizability to type 2 diabetes populations. Many studies were acute in nature, studies with longer intervention duration are needed to assess the impact of bedtime snacks on glycemic control. Furthermore, some studies did not include a no-snack control and therefore could not determine whether a snack is superior to no snack, only which snack is preferred. Varied measures of glycemic control used by the intervention studies also makes comparisons between interventions difficult. Lastly, some interventions implemented such as corn starch before bed or a split dinner are not feasible and therefore lack translation into real-world applications.

In conclusion, this systematic review found no conclusive benefit to consuming a snack before bed for glycemic control in people with type 2 diabetes and prediabetes. More high-quality long-term randomized control trials in this area are warranted to provide further evidence.

## Supplementary Material

nqac245_Supplemental_File

## Data Availability

Data described in the manuscript will not be made available because this manuscript is a systematic review not a meta-analysis, all data discussed has already been presented in Table 1. No analysis was conducted on the data summarized in Table 1.

## Notes

MEF is supported by a National Health and Medical Research Council Investigator Grant. This project received no specific funding from any funding agency or from commercial or not- for-profit sectors.

Supplemental Methods File 1 is available from the “Supplementary data” link in the online posting of the article and from the same link in the online table of contents at https://academic.oup.com/ajcn/.

Abbreviations used: BCAA, branched-chain amino acid; iAUC, incremental AUC; PROSPERO, International Prospective Register of Systematic Reviews.
