# Supplementary material for: Systematic literature review: should a bedtime snack be used to treat hyperglycemia in type 2 diabetes?
Source: Am J Clin Nutr. 2022 Sep 9;116(5):1251–64. doi: 10.1093/ajcn/nqac245 (PMC9630881; doi:10.1093/ajcn/nqac245)
Supplement: nqac245_Supplemental_File [file nqac245_supplemental_file.docx]

***Systematic Literature Review: Should a bedtime snack be used to treat hyperglycaemia in Type 2 Diabetes?***

***Lauren A Roach PhD^1,2^., William Woolfe^1^., Beenu Bastian^1,3^ Elizabeth Neale PhD^1,2^., Monique E Francois PhD^1,2^***

*Supplementary methods file 1: Database search terms*

**Pubmed**

((((((((((((((((("type 2 diabet*"[Text Word]) OR ("insulin Independent type 2 diabet*"[Text Word])) OR ("Type 2 diabetes mellitus"[Text Word])) OR ("type II diabet*"[Text Word])) OR ("insulin Independent type II diabet*"[Text Word])) OR ("Type II diabetes mellitus"[Text Word])) OR ("Diabetes Mellitus, Type 2"[Mesh])) OR (prediabet*[Text Word])) OR ("Prediabetic State"[Mesh])) OR ("non-insulin-dependent diabetes mellitus"[Text Word])) OR (diabet*[Text Word])) OR ("insulin resistan*"[Text Word])) OR ("Insulin Resistance"[Mesh])) OR ("glucose intoleran*"[Text Word])) OR ("impaired glucose toleran*"[Text Word])) OR ("Glucose Intolerance"[Mesh])) AND ((((((((((((("bedtime snack*"[Text Word]) OR ((bedtime[Text Word]) AND (meal*[Text Word]))) OR ((before bed[Text Word]) AND (snack*[Text Word]))) OR ("midnight snack*"[Text Word])) OR ((before bed[Text Word]) AND (meal*[Text Word]))) OR ((midnight[Text Word]) AND (meal*[Text Word]))) OR ("bedtime"[Text Word])) OR ("supper"[Text Word])) OR ("evening"[Text Word])) OR ("night time"[Text Word])) OR ("dinner"[Text Word])) OR ("suppertime"[Text Word])) OR ("bedtime supper"[Text Word]))) AND (((((((((((((((((((((((((((glucose[Text Word]) OR ("Glucose"[Mesh])) OR ("blood glucose"[Text Word])) OR ("Blood Glucose"[Mesh])) OR ("fasting glucose"[Text Word])) OR ("blood sugar*"[Text Word])) OR ("fasting blood glucose"[Text Word])) OR ("fasting blood sugar*"[Text Word])) OR ("glucose tolerance"[Text Word])) OR ("fasting hyperglycaemia"[Text Word])) OR ("fasting hyperglycemia"[Text Word])) OR ("hyperglycaemia"[Text Word])) OR ("hyperglycemia"[Text Word])) OR ("Hyperglycemia"[Mesh])) OR ("insulin"[Text Word])) OR ("Insulin"[Mesh])) OR ("insulin resistance"[Text Word]) OR ("Insulin Resistance"[Mesh])) OR ("HbA1c"[Text Word])) OR ("glycated haemoglobin"[Text Word])) OR ("glycated hemoglobin"[Text Word])) OR ("Glycated Hemoglobin A"[Mesh])) OR ("glycaemic"[Text Word])) OR ("glycemic"[Text Word])) OR ("waking glucose"[Text Word])) OR ("morning hyperglycemia"[Text Word])) OR ("morning hyperglycaemia"[Text Word])) OR ("Dawn phenomenon"[Text Word]))

**Cochrane CENTRAL**

(((((((((((((((((("type 2" NEXT diabet*):ti,ab,kw) OR (("insulin Independent type 2" NEXT diabet*):ti,ab,kw)) OR ("Type 2 diabetes mellitus":ti,ab,kw)) OR (("type II" NEXT diabet*):ti,ab,kw)) OR (("insulin Independent type II" NEXT diabet*):ti,ab,kw)) OR ("Type II diabetes mellitus":ti,ab,kw)) OR ([mh "Diabetes Mellitus, Type 2"])) OR (prediabet*:ti,ab,kw)) OR ([mh "Prediabetic State"])) OR ("non-insulin-dependent diabetes mellitus":ti,ab,kw)) OR (diabet*:ti,ab,kw)) OR (("insulin" NEXT resistan*):ti,ab,kw)) OR ([mh "Insulin Resistance"])) OR (("glucose" NEXT intoleran*):ti,ab,kw)) OR (("impaired glucose" NEXT toleran*):ti,ab,kw)) OR ([mh "Glucose Intolerance"])) AND (((((((((((((("bedtime" NEXT snack*):ti,ab,kw) OR ((bedtime:ti,ab,kw) AND (meal*:ti,ab,kw))) OR (("before bed":ti,ab,kw) AND (snack*:ti,ab,kw))) OR (("midnight" NEXT snack*):ti,ab,kw)) OR (("before bed":ti,ab,kw) AND (meal*:ti,ab,kw))) OR ((midnight:ti,ab,kw) AND (meal*:ti,ab,kw))) OR (bedtime:ti,ab,kw)) OR (supper:ti,ab,kw)) OR (evening:ti,ab,kw)) OR ("night time":ti,ab,kw)) OR (dinner:ti,ab,kw)) OR (suppertime:ti,ab,kw)) OR ("bedtime supper":ti,ab,kw))) AND (((((((((((((((((((((((((((glucose:ti,ab,kw) OR ([mh Glucose])) OR ("blood glucose":ti,ab,kw)) OR ([mh "Blood Glucose"])) OR ("fasting glucose":ti,ab,kw)) OR (("blood" NEXT sugar*):ti,ab,kw)) OR ("fasting blood glucose":ti,ab,kw)) OR (("fasting blood" NEXT sugar*):ti,ab,kw)) OR ("glucose tolerance":ti,ab,kw)) OR ("fasting hyperglycaemia":ti,ab,kw)) OR ("fasting hyperglycemia":ti,ab,kw)) OR (hyperglycaemia:ti,ab,kw)) OR (hyperglycemia:ti,ab,kw)) OR ([mh Hyperglycemia])) OR (insulin:ti,ab,kw)) OR ([mh Insulin])) OR ("insulin resistance":ti,ab,kw) OR ([mh "Insulin Resistance"])) OR (HbA1c:ti,ab,kw)) OR ("glycated haemoglobin":ti,ab,kw)) OR ("glycated hemoglobin":ti,ab,kw)) OR ([mh "Glycated Hemoglobin A"])) OR (glycaemic:ti,ab,kw)) OR (glycemic:ti,ab,kw)) OR ("waking glucose":ti,ab,kw)) OR ("morning hyperglycemia":ti,ab,kw)) OR ("morning hyperglycaemia":ti,ab,kw)) OR ("Dawn phenomenon":ti,ab,kw))

**CINAHL (via EBSCOhost)**

((((((((((((((((("type 2 diabet*") OR ("insulin Independent type 2 diabet*")) OR ("Type 2 diabetes mellitus")) OR ("type II diabet*")) OR ("insulin Independent type II diabet*")) OR ("Type II diabetes mellitus")) OR ((MH "Diabetes Mellitus, Type 2"+))) OR (prediabet*)) OR ((MH "Prediabetic State"+))) OR ("non-insulin-dependent diabetes mellitus")) OR (diabet*)) OR ("insulin resistan*")) OR ((MH "Insulin Resistance"+))) OR ("glucose intoleran*")) OR ("impaired glucose toleran*")) OR ((MH "Glucose Intolerance"+))) AND ((((((((((((("bedtime snack*") OR ((bedtime) AND (meal*))) OR (("before bed") AND (snack*))) OR ("midnight snack*")) OR (("before bed") AND (meal*))) OR ((midnight) AND (meal*))) OR (bedtime)) OR (supper)) OR (evening)) OR ("night time")) OR (dinner)) OR (suppertime)) OR ("bedtime supper"))) AND (((((((((((((((((((((((((((glucose) OR ((MH Glucose+))) OR ("blood glucose")) OR ((MH "Blood Glucose"+))) OR ("fasting glucose")) OR ("blood sugar*")) OR ("fasting blood glucose")) OR ("fasting blood sugar*")) OR ("glucose tolerance")) OR ("fasting hyperglycaemia")) OR ("fasting hyperglycemia")) OR (hyperglycaemia)) OR (hyperglycemia)) OR ((MH Hyperglycemia+))) OR (insulin)) OR ((MH Insulin+))) OR ("insulin resistance") OR ((MH "Insulin Resistance"+))) OR (HbA1c)) OR ("glycated haemoglobin")) OR ("glycated hemoglobin")) OR ((MH "Glycated Hemoglobin A"+))) OR (glycaemic)) OR (glycemic)) OR ("waking glucose")) OR ("morning hyperglycemia")) OR ("morning hyperglycaemia")) OR ("Dawn phenomenon"))

**Medline (via EBSCOhost)**

((((((((((((((((("type 2 diabet*") OR ("insulin Independent type 2 diabet*")) OR ("Type 2 diabetes mellitus")) OR ("type II diabet*")) OR ("insulin Independent type II diabet*")) OR ("Type II diabetes mellitus")) OR ((MH "Diabetes Mellitus, Type 2+”))) OR (prediabet*)) OR ((MH "Prediabetic State”))) OR ("non-insulin-dependent diabetes mellitus")) OR (diabet*)) OR ("insulin resistan*")) OR ((MH "Insulin Resistance+”))) OR ("glucose intoleran*")) OR ("impaired glucose toleran*")) OR ((MH "Glucose Intolerance”))) AND ((((((((((((("bedtime snack*") OR ((bedtime) AND (meal*))) OR (("before bed") AND (snack*))) OR ("midnight snack*")) OR (("before bed") AND (meal*))) OR ((midnight) AND (meal*))) OR (bedtime)) OR (supper)) OR (evening)) OR ("night time")) OR (dinner)) OR (suppertime)) OR ("bedtime supper"))) AND (((((((((((((((((((((((((((glucose) OR ((MH "Glucose+"))) OR ("blood glucose")) OR ((MH "Blood Glucose"))) OR ("fasting glucose")) OR ("blood sugar*")) OR ("fasting blood glucose")) OR ("fasting blood sugar*")) OR ("glucose tolerance")) OR ("fasting hyperglycaemia")) OR ("fasting hyperglycemia")) OR (hyperglycaemia)) OR (hyperglycemia)) OR ((MH "Hyperglycemia+"))) OR (insulin)) OR ((MH "Insulin+"))) OR ("insulin resistance") OR ((MH "Insulin Resistance+"))) OR (HbA1c)) OR ("glycated haemoglobin")) OR ("glycated hemoglobin")) OR ((MH "Glycated Hemoglobin A"))) OR (glycaemic)) OR (glycemic)) OR ("waking glucose")) OR ("morning hyperglycemia")) OR ("morning hyperglycaemia")) OR ("Dawn phenomenon"))
